# Phthalate exposure and its impact on mental health risk in the elderly Thai population

**DOI:** 10.1007/s44192-025-00314-2

**Published:** 2025-12-19

**Authors:** Orawan Kaewboonchoo, Maruth Bhrasartsuwan, Kraichat Tantrakarnapa, Chatchai Ekpanyaskul

**Affiliations:** 1 Department of Public Health Nursing, Faculty of Public Health, Mahidol University, Bangkok, Thailand; 2 Applied Psychology Program, Division of Multi/Interdisciplinary Studies, Graduate School, Srinakharinwirot University, Bangkok, Thailand; 3 Department of Social and Environmental Medicine, Faculty of Tropical Medicine, Mahidol University, Bangkok, Thailand; 4 Department of Preventive and Social Medicine, Faculty of Medicine, Srinakharinwirot University, Bangkok, Thailand

**Keywords:** Mental health, Stress, Anxiety, Depression, Phthalate, Elderly

## Abstract

Phthalates, ubiquitous environmental contaminants, are widely used in daily life. Although their adverse health effects across developmental stages have been well-documented, their impact on elderly populations remains unexplored. This study investigated the association between phthalate exposure and mental health issues (depression, anxiety, and stress). A community-based cross-sectional study was conducted among 688 individuals aged ≥ 60 years in Thailand. Phthalate exposure was assessed using five urinary phthalate metabolites: mono(2-ethylhexyl) phthalate (MEHP), monoisobutyl phthalate, mono-n-butyl phthalate (MnBP), monoethyl phthalate, and monomethyl phthalate. Mental health status was evaluated using the Depression Anxiety Stress Scales (DASS-21), a self-administered questionnaire that assesses depression, anxiety, and stress, along with demographic data. Binary logistic regression adjusted for potential confounders was used to analyze associations with mental health risks. The detection rates for the phthalate metabolites ranged between 31.83 and 96.08%, with MEHP being most frequently detected. Depression emerged as the most prevalent mental health condition. Among healthy participants, those in the highest tertile of urinary MEHP and MnBP levels exhibited significantly increased odds of anxiety, with adjusted odds ratios 3.13 (95% CI 1.37–7.12) and 2.28 (95% CI 1.03–5.06), respectively. Both metabolites exhibited a dose–response relationship (*p*-for-trend = 0.002 and 0.034, respectively). The number needed to harm was 5.66 for MEHP and 8.09 for MnBP. Associations were not observed in individuals with underlying chronic diseases. Furthermore, no association with depression or stress was observed. In conclusion, exposure to both high- and low-molecular-weight phthalates was associated with an increased risk of anxiety among healthy older adults. However, causal inferences were not confirmed owing to the study design. Longitudinal research is warranted to clarify the underlying mechanisms and inform mental health surveillance for older individuals.

## Introduction

Aging, a global public health issue [1], is a period of regressive physiological and psychological function, accompanied by a high incidence of health issues. In addition to physical ailments, mental health issues are a significant concern among elderly individuals. Unlike physical health problems, mental health issues can be mitigated through proactive promotion and prevention strategies. Despite this potential for intervention, mental health disorders remain prevalent and impose a substantial burden on both individuals and society [2, 3]. Mental health issues are a prominent health burden, leading to clinical morbidity, increased mortality, and loss of quality of life, particularly in the elderly population. They also contribute to a significant proportion of disability-adjusted life years [4].

Numerous biological, psychological, and environmental factors affect mental health outcomes. Environmental factors, such as metal exposure, air pollution, pesticides, and noise, are emerging issues that impact health [5, 6]. Phthalates, chemicals commonly used in daily life, have recently gained attention because of their potential health effects [7]. Phthalates are esters of phthalic acid, which are primarily used as plasticizers for enhancing the flexibility and durability of plastics. They are found in food packaging, personal care products, and household items such as flooring and furniture [8–10]. Human exposure to phthalates is almost ubiquitous and varies by underlying chronic disease, sex, ethnicity, and region [11, 12]. They are absorbed through air inhalation, dermal absorption, and dietary sources and are eventually excreted in urine [13].

Phthalates interfere with neuroendocrine function, which can lead to various health issues. As endocrine-disrupting chemicals (EDCs), they interfere with hormonal systems, potentially causing reproductive, developmental, neurological, and immune disorders [14]. Phthalates may affect brain function and contribute to cognitive decline, mood disorders, and other mental health issues [15]. While studies on the effects of phthalates on neuropsychiatric problems during the prenatal, childhood [16], and adolescent periods [17–19] are abundant, their impact on the elderly remains largely unexplored. Elderly individuals are particularly vulnerable because of their compromised physiological resilience and cumulative exposure to environmental toxins [20]. Although animal studies have shown associations between phthalates and mental health [21–26], epidemiological studies in humans are limited, and related studies are necessary to establish a definitive connection.

This study aimed to investigate the association between phthalate exposure and mental health issues in elderly individuals. The objective was to explore how phthalates contribute to depression, anxiety, and stress in the elderly population of Thailand.

## Methods

### Study design

This community-based cross-sectional study was part of the “Project to Eliminate Indoor Dust Pollution for Enhancing the Quality of Life of the Elderly,” approved by the Ethical Committee of Srinakharinwirot University. This study did not involve an intervention or a clinical trial and was conducted in accordance with the Declaration of Helsinki, Belmont Report, and International Ethical Guidelines for Health-related Research Involving Humans of the World Health Organization.

### Participants

Multistage random sampling, including stratified and simple random sampling techniques, was used to determine the areas for data collection. A minimum sample size estimate based on Cohen’s guidelines for multiple regression, assuming a small effect size (f^2^ = 0.02), 90% power, and nine predictors, yielded 535 participants [27]. The number of participants increased by 15% to account for five correlated outcomes, yielding 615 participants [28]. Allowing for a 10% non-response rate, the minimum required sample size for the study was 677 participants. The study covered all regions of Thailand, including Bangkok, the capital city, Phayao and Lopburi provinces in the north, Buriram province in the northeast, Rayong province in the east, and Songkhla province in the south. Elderly individuals from these areas were included, with only one participant selected per household. The inclusion criteria were as follows: 1) individuals aged ≥ 60 years and without a disability, active psychiatric disease, or cognitive problems; 2) residents in their household for > 1 year; and 3) individuals able to participate by reading and answering the questionnaire. All participants provided informed consent prior to data and urine sample collection. Participation was voluntary, and confidentiality was strictly maintained.

### Measures

The data analyzed in this study comprised two components. The first component was a self-administered questionnaire that collected demographic and health-related data, including sex, age group (60–69, 70–79, ≥ 80 years), region of residence, education level, marital and employment status, smoking and alcohol use, and self-reported underlying chronic diseases. Participants were classified as the “healthy elderly group” if they reported no chronic diseases and as the “elderly with underlying chronic disease group” if they had at least one diagnosed chronic disease, such as hypertension, diabetes mellitus, dyslipidemia, or cardiovascular disease. The second component assessed mental health status using the Depression Anxiety Stress Scales (DASS-21) questionnaire [29] previously tested on a community sample [30]. The original version was translated into Thai [31]. DASS-21 comprises 21 questions that evaluate depression, anxiety, and stress. Each dimension contains seven items for measuring each mental health issue over the past two weeks. Examples of items include: for anxiety, "I felt scared without any good reason"; for depression, "I found it difficult to work up the initiative to do things"; and for stress, "I was intolerant of anything that kept me from getting on with what I was doing." Each item is rated on a 4- point rating scale ranging between 0 and 3 (0 = did not apply to me at all; 1 = applied to me to some extent or some of the time; 2 = applied to me to a significant extent or a good part of the time; 3 = applied to me very much or most of the time). The total score for each subscale was calculated separately, and the sum of scores for the seven items in each category was used. Based on the Thai version of DASS-21, the thresholds defined abnormal levels as ≥ 4 for depression, ≥ 3 for anxiety, and ≥ 7 for stress.

DASS-21 has been used in different countries, and its reliability and validity have been confirmed [31]. Cronbach’s alpha coefficients of depression, anxiety, and stress in Thailand are 0.82, 0.78, and 0.69, respectively [32]. Moreover, the correlation coefficients of the test–retest reliability of DASS-21 scores for depression, anxiety, and stress in a pilot study involving 30 Thai elderly participants were 0.623, 0.843, and 0.809, respectively.

For phthalate exposure, this study focused on five phthalate metabolites that are among the most abundant compounds found in urine [33] and are subject to regulatory control in Thailand. The metabolites analyzed included mono(2-ethylhexyl) phthalate (MEHP) metabolite of di(2-ethylhexyl) phthalate (DEHP), monoisobutyl phthalate (MiBP) metabolite of diisobutyl phthalate (DiBP), mono-n-butyl phthalate (MnBP) metabolite of di-n-butyl phthalate (DnBP), monoethyl phthalate (MEP) metabolite of diethyl phthalate (DEP), and monomethyl phthalate (MMP) metabolite of dimethyl phthalate (DMP).

Urine samples were collected by trained local health volunteers on the same day as the questionnaire data were obtained. Spot urine samples of at least 10 cc were collected in the morning from each elderly participant using phthalate-free polypropylene tubes (Nunc™, Thermo Fisher Scientific, Waltham, MA, USA). The samples were stored at -20 °C until analysis and sent to a laboratory for high-performance liquid chromatography–electrospray ionization-tandem mass spectrometry. The method for analyzing urinary metabolites was adapted from the laboratory procedure manual of Emory University, Rollins School of Public Health [34]. Prior to preparation, samples were thawed at room temperature (25 °C). A standard stock solution for each analyte was prepared at 400 µg/mL in acetonitrile and stored at − 20 °C. To each sample, 1 mL of 2000 U/mL β-glucuronidase/sulfatase in 1 mM ammonium acetate buffer (pH 5) was added and vortexed. The samples were rotated at 150 rpm overnight at 37 °C (approximately 15 h). After incubation, 1.5 mL of 0.15 M sodium phosphate buffer (pH 2) was added to terminate the reaction. The samples were then loaded onto conditioned ABS-Elut-NEXUS (60 mg, 3 cc) solid-phase extraction cartridges and pre-washed with acetonitrile and sodium phosphate buffer. The cartridges were washed with 0.1 M formic acid and Bisphenol A (BPA)-free water before being dried under negative pressure. Samples were eluted with acetonitrile and ethyl acetate and then evaporated in a TurboVap at 50 °C. Dry samples were reconstituted with 100 μL BPA-free water and transferred to autosampler vials with glass inserts.

Calibration solutions for MEHP, MiBP, MnBP, MEP, and MMP were prepared in blank urine at concentrations ranging between 0.01 and 10 μg/mL and treated by the same procedure as that for the urine samples before analysis. A sample preparation run included non-matrix calibrants, quality controls, a reagent blank, and unknown samples. Target analytes were separated by liquid chromatography and analyzed using negative-mode electrospray ionization tandem mass spectrometry. The total run time was 27 min with a 5 μL injection volume using a Betasil Phenyl column (3 μm, 2.1 mm, 150 mm). The mobile phase consisted of acetic acid in BPA-free water and acetonitrile, with a flow rate of 0.3 mL/min and a maximum pressure of 500 bar. Calibration standards showed linearity with a correlation coefficient of > 0.99.

The limit of detection (LOD) for the five metabolites was 0.01 µg/L. For concentrations below the LOD, the values were substituted by the LOD divided by the square root of 2, a method commonly used in environmental health research [34]. All results were adjusted and reported in micrograms per gram of creatinine (µg/g Cr) to standardize for variability in spot urine sample [35].

### Statistical analysis

The data were assessed for missing values and consistency and then analyzed using SPSS v.26.0 (IBM Corp.). Descriptive statistics included frequencies and percentages for categorical variables and mean, standard deviation (SD), minimum, maximum, and median for continuous variables. Urinary metabolite levels were reported as the geometric mean (GM) and geometric SD (GSD). The prevalence and 95% confidence intervals (CIs) of abnormal mental health (depression, anxiety, and stress) were assessed using DASS cutoff scores. Phthalate metabolites were divided into tertiles, with the lowest tertile serving as the reference. Associations with mental health outcomes were analyzed using multiple logistic regression, presenting crude and adjusted odds ratios (aORs) with 95% CIs. Covariates included sex, age, region, education, marital status, employment, smoking, alcohol use, and underlying chronic diseases. The regression assumptions were verified, including independence of observations and the absence of multicollinearity, as indicated by variance inflation factors less than 2.0. Risk differences were also reported in the number needed to harm (NNH), which provides a clinically interpretable measure of effect size. The interaction between underlying chronic diseases and metabolite levels was tested, and stratified analyses were conducted accordingly. Dose–response trends were evaluated using the chi-squared test. Statistical significance was set at *p* < 0.05.

## Results

A total of 688 participants completed the questionnaire and consented to providing a urine sample, resulting in a participation rate of 71%. Their ages ranged between 60 and 95 years, with a mean age of 70.42 ± 7.83 years, and the male-to-female ratio was 1:2. Most participants were married and had an educational level of primary school or below. Nearly 50% were still working. Additionally, 7% were active smokers, and 9.9% were active drinkers. Approximately 59.2% had underlying chronic diseases such as hypertension (46.8%), diabetes mellitus (22.2%), heart disease (6.8%), or cancer (1.6%). The detailed demographic information is presented in Table 1.

**Table 1:** General characteristics of elderly participants and prevalence of each dimension of mental health issues across study factors

Characteristics (n = 688)	Number (%)	Prevalence of each mental health issue		
		Depression	Anxiety	Stress
Sex		*p* = 0.316	*p* = *0.528*	*p* = *0.239*
Male	228 (33.1)	39.04	22.81	10.53
Female	460 (66.9)	43.04	25.00	13.70
Age group (years)		*p* = 0.630	*p* = *0.467*	*p* = *0.070*
60–69	363 (52.8)	41.05	24.24	11.85
70–79	216 (31.4)	40.74	22.22	10.65
> 80	109 (15.8)	45.87	28.44	19.27
Region		*p* < 0.001	*p* < 0.001	*p* = *0.002*
Capital city	108 (15.7)	34.26	17.59	11.11
North	166 (24.1)	30.12	10.24	4.82
Northeast	77 (11.2)	29.87	24.68	9.09
East	162 (23.6)	50.00	31.48	18.52
South	175 (25.4)	54.86	34.86	17.14
Education level		*p* = 0.157	*p* = 0.321	*p* = *0.264*
Primary school or under	468 (68.0)	40.38	25.21	13.46
Secondary school	126 (18.3)	49.21	25.40	13.49
Vocational or bachelor degree or upper	94 (13.7)	38.30	18.09	7.45
Marital status		*p* = 0.911	*p* = 0.211	*p* = *0.230*
Single	44 (6.4)	40.91	13.64	4.55
Married	382 (55.5)	41.10	24.35	13.61
Divorce or widow	262 (38.1)	42.75	25.95	12.60
Employment status		*p* = 0.002	*p* = 0.347	*p* = *0.842*
Retried	349 (50.7)	47.56	25.79	12.89
Still working	339 (49.3)	35.69	22.71	12.39
Smoking status		*p* = 0.354	*p* = 0.243	*p* = *0.895*
Non-smoker	588 (85.5)	40.65	23.98	12.59
Ex-smoker	52 (7.5)	50.00	32.69	11.54
Active smoker	48 (7.0)	45.83	18.75	14.58
Alcohol status		*p* = 0.203	*p* = 0.192	*p* = *0.070*
Non-drinker	561(81.5)	41.53	25.67	13.90
Ex-drinker	59 (8.6)	50.85	16.95	10.17
Active drinker	68 (9.9)	35.29	19.12	4.41
Underlying chronic disease		*p* = 0.507	*p* = 0.027	*p* = *0.197*
No	281 (40.8)	40.21	19.93	10.68
Yes	407 (59.2)	42.75	27.27	14.00

The overall prevalence rates of depression, anxiety, and stress were 41.72% (95% CI = 38.02–46.41%), 24.27% (95% CI = 21.06–27.48%), and 12.65% (95% CI = 10.16–15.14%), respectively. The prevalence rates stratified by each demographic data are presented in Table 1.

The detection rates of the five phthalate metabolites in urine above the LOD ranged between 31.83 and 96.08%. The prevalence of the detected phthalate metabolites from highest to lowest was as follows: MEHP, MEP, MiBP, MnBP, and MMP. Descriptive statistics for the concentrations of these five metabolites are shown in Table 2.

**Table 2:** Descriptive statistics of the phthalate metabolites in urine samples of elderly participants (n = 688)

Urinary metabolites	Number of participants with detection above LOD level (%)	Minimum (µg/g Cr)	Maximum (µg/g Cr)	Mean (µg/g Cr)	Standard deviation (µg/g Cr)	Median (µg/g Cr)	Geometric mean (µg/g Cr)	Geometric standarddeviation (µg/g Cr)
MEHP	661 (96.08)	< 0.01	2123.43	143.39	198.47	80.72	1.73	0.95
MiBP	538 (78.20)	< 0.01	1882.89	48.24	94.73	25.37	0.68	1.59
MnBP	506 (73.55)	< 0.01	11,748.31	283.19	970.37	53.44	0.93	1.86
MEP	538 (78.20)	< 0.01	8302.21	434.66	1020.68	77.26	1.22	1.90
MMP	219 (31.83)	< 0.01	3932.59	68.96	266.57	0.01	0.74	1.88

The results of the association analysis between each metabolite and every mental health issue after adjusting for possible confounders, including sex, age group, region of residence, educational level, marital status, employment status, smoking and alcohol behavior, and underlying chronic diseases, indicated no associations. However, a significant interaction was observed; when stratified by underlying chronic disease and found that a significant association between the highest tertile of urinary MEHP and anxiety was identified only in the healthy elderly group, with an aOR of 3.126 (95% CI 1.372–7.120), with a *p*-value for trend of 0.002, indicating a dose–response relationship, and an NNH of 5.66 (95% CI 3.48–15.08). Additionally, an association between the highest tertile of urinary MnBP and anxiety was observed in the healthy elderly, with an aOR of 2.279 (95% CI 1.026–5.059), with a *p*-value for trend of 0.034, and an NNH of 8.09 (95% CI 4.27–77.52). However, the association was not significant in elderly individuals experiencing depression or stress. Moreover, no significant associations were observed with any mental health problems in patients with underlying chronic diseases. (Tables 3, 4 and 5).

**Table 3:** Association between urinary phthalate metabolites and depression among all participants, healthy elderly, and elderly with underlying chronic diseases

Urinary metabolites	All participants (n = 688)		Heathy elderly (n = 281)		Elderly with underlying chronic diseases (n = 407)	
	CrudeORs (95% CI)	AdjustedORs (95%CI)^a^	Crude ORs (95% CI)	Adjusted ORs (95% CI)^b^	Crude ORs (95% CI)	Adjusted ORs (95% CI)^b^
*MEHP*						
Tertile 1	Reference	Reference	Reference	Reference	Reference	Reference
Tertile 2	0.76(0.52–1.11)	0.79(0.54–1.17)	0.96(0.53–1.72)	1.03(0.56–1.89)	0.75(0.45–1.25)	0.72(0.44–1.18)
Tertile 3	1.09(0.76–1.58)	1.12(0.77–1.65)	1.10(0.62–1.97)	1.09(0.59–2.01)	1.14(0.69–1.89)	1.17(0.72–1.88)
*MiBP*						
Tertile 1	Reference	Reference	Reference	Reference	Reference	Reference
Tertile 2	1.07 (0.74–1.56)	1.13(0.77–1.65)	0.88(0.49–1.57)	0.88(0.49–1.61)	1.19(0.73–1.92)	1.28(0.77–2.11)
Tertile 3	0.78(0.54–1.13)	0.91(0.62–1.33)	0.62(0.34–1.11)	0.58(0.31–1.08)	1.14(0.71–1.85)	1.13(0.69–1.87)
*MnBP*						
Tertile 1	Reference	Reference	Reference	Reference	Reference	Reference
Tertile 2	1.10(0.75–1.61)	1.10(0.75–1.61)	0.92(0.51–1.64)	0.86(0.47–1.58)	1.15(0.71–1.85)	1.16(0.70–1.92)
Tertile 3	0.83(0.56–1.22)	0.83(0.56–1.22)	0.68(0.38–1.22)	0.67(0.36–1.25)	0.82(0.50–1.33)	0.88(0.53–1.45)
*MEP*						
Tertile 1	Reference	Reference	Reference	Reference	Reference	Reference
Tertile 2	1.10(0.75–1.59)	1.06(0.72–1.56)	0.84(0.47–1.50)	0.79(0.43–1.45)	1.42(0.88–2.31)	1.40(0.84–2.33)
Tertile 3	1.00(0.69–1.45)	0.99(0.68–1.46)	0.85(0.47–1.51)	0.81(0.44–1.49)	1.14(0.70–1.86)	1.18(0.71–1.96)
*MMP*						
Tertile 1 & 2	Reference	Reference	Reference	Reference	Reference	Reference
Tertile 3	0.89(0.64–1.23)	0.87(0.62–1.22)	0.79(0.47–1.33)	0.81(0.47–1.37)	0.96(0.63–1.45)	0.91(0.59–1.41)

a Adjusted for sex, age group, region of residence, education, marital status, employment status, smoking status, alcohol status, and underlying chronic diseases

b Adjusted for sex, age group, region of residence, education, marital status, employment status, smoking status, and alcohol status

**Table 4:** Association between urinary phthalate metabolites and anxiety among all participants, healthy elderly, and elderly with underlying chronic diseases

Urinary metabolites	All participants (n = 688)		Heathy elderly (n = 281)		Elderly with underlying chronic diseases (n = 407)	
	Crude ORs (95% CI)	Adjusted ORs (95% CI)^a^	Crude ORs (95% CI)	Adjusted ORs (95% CI)^b^	Crude ORs (95% CI)	Adjusted ORs (95% CI)^b^
*MEHP*						
Tertile 1	Reference	Reference	Reference	Reference	Reference	Reference
Tertile 2	0.97(0.62–1.52)	0.92(0.58–1.46)	2.13(0.93–4.87)	2.29(0.98–5.35)	0.75(0.44–1.31)	0.75(0.42–1.32)
Tertile 3	1.45(0.95–2.21)	1.44(0.93–2.23)	3.30(1.49–7.29)	3.13(1.37–7.12)	1.11(0.66–1.86)	1.10(0.64–1.88)
*MiBP*						
Tertile 1	Reference	Reference	Reference	Reference	Reference	Reference
Tertile 2	1.23(0.79–1.91)	1.27(0.81–1.99)	1.69(0.78–3.63)	1.68(0.76–3.68)	1.07(0.62–1.84)	1.10(0.63–1.91)
Tertile 3	1.47(0.96–2.26)	1.49(0.96–2.30)	1.97(0.93–4.17)	1.90(0.88–4.13)	1.24(0.73–2.12)	1.25(0.72–2.15)
*MnBP*						
Tertile 1	Reference	Reference	Reference	Reference	Reference	Reference
Tertile 2	1.42(0.91–2.21)	1.43(0.91–2.24)	1.85(0.85–4.04)	1.72(0.77–3.85)	1.34(0.78–2.31)	1.34(0.77–2.33)
Tertile 3	1.49(0.96–2.30)	1.55(0.99–2.42)	2.28(1.06–4.89)	2.28(1.03–5.06)	1.26(0.73–2.16)	1.31(0.76–2.29)
*MEP*						
Tertile 1	Reference	Reference	Reference	Reference	Reference	Reference
Tertile 2	1.28(0.83–1.98)	1.31(0.84–2.05)	0.93(0.45–1.95)	0.95(0.44–2.02)	1.62(0.94–2.79)	1.61(0.92–2.82)
Tertile 3	1.32(0.85–2.03)	1.28(0.83–1.99)	1.18(0.58–2.40)	1.14(0.55–2.37)	1.32(0.76–2.29)	1.33(0.76–2.35)
*MMP*						
Tertile 1 & 2	Reference	Reference	Reference	Reference	Reference	Reference
Tertile 3	0.92(0.63–1.35)	0.94(0.64–1.38)	1.28(0.69–2.38)	1.40(0.74–2.64)	0.76(0.47–1.23)	0.75(0.46–1.22)

a Adjusted for sex, age group, region of residence, education, marital status, employment status, smoking status, alcohol status, and underlying chronic diseases

b Adjusted for sex, age group, region of residence, education, marital status, employment status, smoking status, and alcohol status

**Table 5:** Association between urinary phthalates metabolites and stress among all participants, healthy elderly, and elderly with underlying chronic disease

Urinary metabolites	All participants (n = 688)		Heathy elderly (n = 281)		Elderly with underlying chronic diseases (n = 407)	
	Crude ORs (95% CI)	Adjusted ORs (95% CI)^a^	Crude ORs (95% CI)	Adjusted ORs (95% CI)^b^	Crude ORs (95% CI)	Adjusted ORs (95% CI)^b^
*MEHP*						
Tertile 1	Reference	Reference	Reference	Reference	Reference	Reference
Tertile 2	0.71(0.39–1.26)	0.68(0.37–1.24)	1.32(0.47–3.70)	1.41(0.49–4.10)	0.54(0.26–1.13)	0.50(0.24–1.07)
Tertile 3	1.16(0.68–1.95)	1.18(0.68–2.04)	2.12(0.82–5.53)	2.10(0.77–5.71)	0.97(0.51–1.84)	0.99(0.51–1.95)
*MiBP*						
Tertile 1	Reference	Reference	Reference	Reference	Reference	Reference
Tertile 2	1.33(0.76–2.32)	1.28(0.72–2.26)	1.12(0.44–2.91)	0.98(0.37–2.64)	1.72(0.86–3.44)	1.68(0.83–3.42)
Tertile 3	1.19(0.68–2.09)	1.16(0.65–2.06)	1.22(0.48–3.10)	1.06(0.40–2.82)	1.19(0.57–2.47)	1.15(0.55–2.43)
*MnBP*						
Tertile 1	Reference	Reference	Reference	Reference	Reference	Reference
Tertile 2	1.22(0.70–2.13)	1.24(0.70–2.19)	1.32(0.47–3.70)	1.23(0.42–3.57)	1.49(0.76–2.92)	1.51(0.75–3.02)
Tertile 3	1.14(0.65–1.99)	1.09(0.61–1.93)	2.12(0.82–5.53)	1.74(0.64–4.76)	0.90(0.44–1.87)	0.88(0.42–1.86)
*MEP*						
Tertile 1	Reference	Reference	Reference	Reference	Reference	Reference
Tertile 2	1.30(0.73–2.32)	1.28(0.71–2.32)	1.00(0.36–2.79)	0.96(0.33–2.74)	1.95(0.96–3.94)	1.89(0.91–3.92)
Tertile 3	1.56(0.89–2.73)	1.46(0.83–2.58)	1.84(0.73–4.61)	1.52(0.58–3.96)	1.29(0.61–2.70)	1.20(0.56–2.56)
*MMP*						
Tertile 1 & 2	Reference	Reference	Reference	Reference	Reference	Reference
Tertile 3	0.96(0.59–1.56)	1.02(0.62–1.66)	1.54(0.71–3.34)	1.62(0.72–3.62)	0.72(0.38–1.35)	0.75(0.39-1.42)

a Adjusted for sex, age group, region of residence, education, marital status, employment status, smoking status, alcohol status, and underlying chronic diseases

b Adjusted for sex, age group, region of residence, education, marital status, employment status, smoking status, and alcohol status

## Discussion

In this community-based cross-sectional study conducted across all regions of Thailand, a significant association was observed between the highest tertile of urinary MEHP levels and an increased risk of anxiety in healthy elderly individuals. Similarly, the highest tertile of urinary MnBP levels and anxiety risk in this demographic exhibited an association. Notably, these associations were not observed in individuals with underlying health conditions.

Phthalate is an emerging environmental toxin of global concern [6, 7], with evidence demonstrating its effects on physical health [36]; however, limited research has been conducted on its effects on psychological health, particularly in the elderly. Thailand reports one of the highest prevalences of mental health issues in Asia, as assessed using the DASS-21 tool [37]. However, the magnitude of mental health issues among the elderly varies by region and situation [38, 39]. For example, this study indicated the highest rates were in the southern region, with cultural or environmental factors likely contributing to these differences. The prevalence of mental health issues is higher among females than males, possibly owing to social roles, contexts, and systemic factors [40].

MEHP serves as a surrogate for DEHP metabolites, indicating exposure to high molecular-weight phthalates, which is highest among the elderly Thai population [41]. Conversely, MnBP is a surrogate for DnBP metabolites and indicates exposure to low molecular-weight phthalates. Phthalates are diesters that are initially hydrolyzed into their respective monoesters, which can then undergo further modifications through oxidation reactions. Both hydrolytic monoesters and oxidized metabolites conjugate with glucuronic acid, and the majority are secreted through urine. These compounds have recently raised concerns owing to their potential neurotoxic effects. The results of this study showed that high exposure to both MEHP and MnBP increased anxiety risk.

Anxiety is increasingly prevalent in the elderly, characterized by heightened and persistent responses to perceived threats. The association between MEHP, MnBP, and anxiety aligns with several Bradford–Hill Criteria for causation [42], as follows:

1. Strength of the association: The observed adjusted odds ratios and 95% CIs for MEHP and MnBP were > 1.0, indicating a risk association. The strength of the associations ranging between 1.03 and 7.12 suggests the likelihood of a real effect, particularly when accompanied by statistically significant trends for some phthalates (*p*-for-trend < 0.05).
2. Consistency of evidence: Since this is one of the few epidemiological studies on mental health associations [17], consistency with findings from other human studies remains undetermined. However, the consistency between the findings of this study and those of different lifespan populations strengthens the likelihood of an effect. Moreover, animal studies also support these findings [21–26, 43].
3. Specificity: While anxiety may be caused by various factors, such as underlying chronic diseases, employment status, and demographic data [38, 44], this study found a specific association only for healthy individuals. Moreover, the results revealed an association between phthalates and only one dimension of mental health issues, in the same direction. However, the specificity criterion makes it unlikely that phthalates are the sole cause of any single non-communicable disease.
4. Temporal relationship: This issue remains a key limitation owing to the cross-sectional nature of this study. Establishing whether phthalate exposure precedes the onset of anxiety is essential for inferring causality.
5. Biological gradient or dose–response relationship: A significant *p*-value for the trend observed for MEHP and MnBP suggests a dose–response relationship, which is an important indicator of causality. Relatively high levels of urinary metabolites, which indicate high phthalate exposure, are associated with increased odds of anxiety, strengthening the causal link. This finding is consistent with those of other studies indicating an association at high doses and high toxicity, as evidenced by behavioral impairments compared to those at low doses leading to reduced anxiety-like behavior [45].
6. Plausibility: Animal studies demonstrating a link between phthalates and anxiogenic effects provide biological plausibility in older individuals; phthalates may aggravate existing age-related vulnerabilities and influence neurotransmitters, neuroinflammation, cognitive function, and hormonal balance, all of which can collectively increase the risk of anxiety. Phthalates disrupt neuroendocrine function, affecting neurological systems through mechanisms such as oxidative stress [23], and inflammatory mediators play a pivotal role in DEHP-induced neurobehavioral impairment [46]. The plausibility that phthalates contribute to anxiety is strong.
7. Coherence: This study presents a coherent picture across different models. The findings align with those of animal studies [21–26] reporting similar neuropsychiatric effects and dose–response patterns. For instance, medium and high doses of MEHP are associated with anxiety [45], whereas MnBP shows such effects only at high doses [43]. These results are consistent with the broad scientific understanding of the effects of EDCs on mental health [47].
8. Experimental evidence: Numerous animal studies have provided experimental evidence supporting an association between phthalates and anxiety [21–26, 43]. Those evidences can be considered analogous to the human experimental data, reinforcing the plausibility of a causal link. However, experimental evidence from animal models may not satisfy the experimental criteria of human trials.
9. Analogous evidence: The analogy criterion supports the case for phthalates because they are known neuroendocrine disruptors [47]. Their mechanism of action is similar to that of other endocrine-disrupting chemicals, such as BPA, which have been linked to neuropsychological outcomes, such as anxiety. This similarity lends credibility to the observed effects of phthalates.

Overall, this study demonstrated a strong association, a dose–response relationship, biological plausibility, and coherence with existing animal studies, suggesting that phthalate exposure may be linked to anxiety, particularly in healthy elderly individuals. However, the lack of temporality and reliance on animal data underscore the need for further longitudinal epidemiological studies to establish a definitive causal relationship.

Healthy elderly individuals may possess intact and responsive neuroendocrine systems—particularly the hypothalamic–pituitary–adrenal axis—potentially making them more susceptible to phthalate-induced dysregulation [47]. Phthalate metabolites, such as MEHP and MnBP, act as EDCs, which interfere with glucocorticoid receptor signaling and cortisol homeostasis, increasing allostatic load and vulnerability to anxiety. These compounds may also induce oxidative stress and neuroinflammation and alter neurotransmitters, such as gamma-aminobutyric acid and serotonin, which directly regulate anxiety [23]. However, the lack of an association with depression and stress in this group may be because of differing pathophysiological pathways, relatively high psychosocial resilience, or distinct neurochemical mechanisms that are less affected by EDCs, suggesting relatively highly specific vulnerability to anxiety-related processes. Additionally, the lack of an association between MEHP and MnBP with anxiety in elderly individuals suffering from underlying chronic diseases may be due to a ceiling effect. The prevalence of anxiety is higher in elderly individuals with underlying chronic diseases than in healthy individuals, which could mask low variable anxiety effects, and detecting the impact of phthalate exposure becomes difficult [48].

## Limitations and suggestions

These results support the routine monitoring of phthalate exposure as a preventative measure for mental health issues. This study had some limitations. First, mental health outcomes may be influenced by various confounding factors such as demographic data and underlying chronic diseases. This study sought to mitigate this issue by adjusting for potential confounders through multiple logistic regression analysis, including demographic data in the model. Second, the mental health outcomes were based on thresholds for regional Thai populations, which differed from the original DASS. Additionally, the self-evaluated DASS-21 is a validated screening tool but is not a clinical diagnostic measure; therefore, its use may introduce misclassifications. However, the alternating item arrangement of the questionnaire minimized this risk, and the assessors did not notice unidirectional bias. Future studies should explore a broad range of mental health outcomes using repeated measures to address reverse causation. Finally, phthalate exposure stems from multiple sources, including indoor environments and personal consumer products, through inhalation, dermal absorption, or ingestion. After exposure, phthalates are metabolized and excreted in urine. Urinary concentrations can vary depending on the hydration status [49] or certain demographic factors, such as body mass index, which were not assessed in this study [50]. Consequently, the capacity to identify the precise sources of exposure was limited. Notably, dust samples should be collected from the households of elderly participants to provide evidence of indoor exposure issues.

This study highlighted the critical link between environmental hazards, such as phthalates, and mental health in the elderly, emphasizing the need for prevention and intervention strategies. Identifying these associations is essential for minimizing phthalate exposure to protect the mental health of older populations. Phthalate exposure is a potentially modifiable risk factor, which can be addressed by lifestyle and public policy changes, such as promoting phthalate-free products and healthy home environments. These findings can inform regulatory measures for reducing phthalate exposure and guide healthcare providers in addressing environmental risks for mental health benefits. Ultimately, promoting healthy aging could alleviate the social and economic burdens of mental health disorders in the elderly.

## Conclusions

As the human lifespan increases, age-related physical health issues become inevitable, whereas mental health challenges remain modifiable through targeted interventions. Environmental factors, such as phthalate exposure, significantly influence mental health outcomes, particularly in healthy elderly individuals. This epidemiological study highlights the association between exposure to high- and low-molecular-weight phthalates and an increased risk of anxiety. Although the observational design limits causal inference, the findings emphasize the need for further longitudinal studies for clarifying the underlying mechanisms. Understanding these associations is critical for developing future strategies to support mental well-being in the aging population, and ongoing surveillance for environment-related community mental health prevention is necessary.

## Data Availability

The data utilized and analyzed in this study are available on reasonable request from the corresponding author.

## Abbreviations

EDC: Endocrine-disrupting chemicals
DASS: Depression anxiety stress scales
MEHP: Mono(2-ethylhexyl) phthalate
DEHP: Di(2-ethylhexyl) phthalate
MiBP: Monoisobutyl phthalate
DiBP: Diisobutyl phthalate
MnBP: Mono-n-butyl phthalate
DnBP: Di-n-butyl phthalate
MEP: Monoethyl phthalate
DEP: Diethyl phthalate
MMP: Monomethyl phthalate
DMP: Dimethyl phthalate
BPA: Bisphenol A
LOD: Limit of detection
SD: Standard deviation
GM: Geometric mean
GSD: Geometric standard deviation
aORs: Adjusted odds ratios
95% CIs: 95% Confidence intervals
NNH: Number needed to harm
